# Vitamin D-Binding Protein (Gc-Globulin) in Acute Liver Failure in Children

**DOI:** 10.3390/diagnostics10050278

**Published:** 2020-05-04

**Authors:** Alina Grama, Lucia Burac, Cornel Olimpiu Aldea, Bogdan Bulata, Dan Delean, Gabriel Samasca, Carmen Abrudan, Claudia Sirbe, Tudor Lucian Pop

**Affiliations:** 1 2nd Paediatric Clinic, University of Medicine and Pharmacy “Iuliu Hatieganu”, 400177 Cluj-Napoca, Romania; gramaalina16@yahoo.com (A.G.); lucabu@hotmail.com (L.B.); claudia.sirbe@yahoo.com (C.S.); 2 Centre of Expertise in Paediatric Liver Rare Diseases, 2nd Paediatric Clinic, Emergency Clinical Hospital for Children, 400177 Cluj-Napoca, Romania; 3 University of Medicine and Pharmacy “Carol Davila”, 050474 Bucharest, Romania; 4 Paediatric Nephrology, Dialysis and Toxicology Clinic, Emergency Clinical Hospital for Children, 400177 Cluj-Napoca, Romania; cornelaldea65@yahoo.com (C.O.A.); bogdan.bulata@yahoo.com (B.B.); ddelean2003@yahoo.com (D.D.); 5 Laboratory of Medical Analysis, Emergency Clinical Hospital for Children, 400177 Cluj-Napoca, Romania; samascagabriel@yahoo.com (G.S.); carmenabrudan@yahoo.com (C.A.)

**Keywords:** acute liver failure, children, Gc-globulin, vitamin D binding protein, prognostic marker, liver transplant, mortality

## Abstract

This study aimed to analyse vitamin D-binding protein (Gc-globulin) serum levels in acute liver failure (ALF) in children in relation to disease outcomes and correlations with other known markers used to evaluate the severity of ALF. Our study included 34 children (mean age 4.87 ± 5.30 years) with ALF of different causes (metabolic, 26.47%; autoimmune, 23.53%; toxic, 20.59%; infection, 17.65%; unknown, 11.76%) and 30 children without any liver injury (mean age 6.11 ± 4.26 years). The outcome was poor in 14 patients (41.18%), including one child with liver transplantation (2.94%). Serum Gc-globulin levels were significantly lower in ALF patients compared to the control group (151.57 ± 171.8 mg/L vs. 498.63 ± 252.50 mg/L; *p* < 0.000001), with an optimum cut-off of 163.5 mg/L (Area Under the Curve, AUC, 0.8921; sensitivity, 76.50%; specificity, 100%). Levels were also lower in patients with poor outcomes compared to survivors (59.34 ± 33.73 mg/L vs. 216.12 ± 199.69 mg/L; *p* < 0.0001), with an optimum cut-off 115 mg/L (AUC, 0.7642; sensitivity, 100%; specificity, 50%). Gc-globulin serum levels were variable according to ALF aetiology, i.e., lower in metabolic, infectious, or unknown causes compared to autoimmune and toxic causes. Gc-globulin serum levels were decreased in children with ALF and lower in those with poor outcomes compared with survivors. Gc-globulin serum levels were correlated with other known parameters used to evaluate the severity of ALF and could help to identify patients at high risk for poor outcomes.

## 1. Introduction

Paediatric acute liver failure (ALF) is a severe condition defined according to the Paediatric Acute Liver Failure study group (PALFSG) criteria as a moderate coagulopathy (International Normalized Ratio, INR 1.5–1.9) that cannot be corrected with parenteral vitamin K in the presence of encephalopathy, or severe coagulopathy (INR > 2.0) in the absence of encephalopathy, in a previously healthy child [1,2,3].

Clinical and laboratory parameters are used to predict the evolution of ALF in children, such as encephalopathy, INR or coagulation factors V or VII [4,5,6,7,8]. King’s College criteria and the Paediatric End-Stage Liver Disease (PELD) and Model for End-Stage Liver Disease (MELD) scores can be used to determine prognosis for the evolution of patients with ALF [9,10,11,12,13,14,15].

Previous studies presented the role of new biological markers in predicting the evolution of ALF in adults. Gc-globulin serum levels were previously used to identify adults with high risk of fatal evolution, with different studies revealing that Gc-globulin levels were significantly lower in patients who died compared to those who survived. In ALF, Gc-globulin levels drop rapidly as the liver synthesises it, with some authors reporting concentrations that were 75% lower compared with adults without ALF [16,17,18,19].

Our study aimed to assess serum levels of Gc-globulin in children with ALF and identify correlations with disease outcomes and other factors used to assess disease severity, namely, coagulation factors V and VII, INR, PELD score, and King’s College criteria.

## 2. Materials and Methods

### 2.1. Patient Enrolment

We enrolled 34 children hospitalised with ALF in this prospective study, who were followed-up in the 2nd Paediatric Clinic of the Emergency Clinical Hospital for Children, Cluj-Napoca, Romania, over a period of four years (January 2015–December 2018), alongside 30 children without liver illness (the control group). We used the PALFSG criteria to select the ALF patients included in this study, including those with jaundice, coagulopathy unable to be corrected by vitamin K infusion (INR > 1.5–1.9) and liver encephalopathy or INR > 2.0 without encephalopathy in a patient with no pre-existing liver condition [1,2,3].

This study was carried out based on approval issued by the Ethics Committee of the Emergency Clinical Hospital for Children in Cluj-Napoca, Romania (41 SC/11.01.2016), after receiving informed consent from the parent/guardian.

### 2.2. Study Procedures

Demographic data was obtained from patient observation charts, which met the PALFSG criteria in terms of ALF, and from charts drawn up for the children in the reference group. We analysed the demographic characteristics of the patients (age, gender, weight), the specific lab tests carried out for severe liver injury upon patient admission to hospital (transaminases, total and direct bilirubin, albumin, INR, prothrombin time and prothrombin%), the PELD score calculated on the first day of hospitalisation and diagnostic and specific investigations to determine the aetiology and evolution of each case (survival, transplant or death).

### 2.3. Measurement of Serum Markers

Total Gc-globulin serum levels were measured in the medical lab of the Emergency Clinical Hospital for Children in Cluj-Napoca, Romania. Biological samples were collected upon patient admission to hospital. Patients’ sera were centrifuged right after collection in the lab, then kept at −200 °C. Gc-globulin was analysed using a Vitamin D-Binding Protein ELISA Kit (VDPB), DRG ELISA 5088 (DRG International, Inc., Springfield, NJ, USA) following the manufacturer’s recommendations and the instructions stated in the user manuals. Coagulation factors V and VII were dosed by Bioclinica Laboratories, Romania, using a coagulometric method. This method consisted of measuring the sample coagulation time, with the sample processed after it was placed in contact with thromboplastin and plasma containing excess coagulation plasma factors other than factors V or VII [20].

### 2.4. Data Analysis and Statistics

All obtained data were included in a database set up in Microsoft Office Excel and statistically interpreted using the Statistica software, Version 13, TIBCO Software Inc., Palo Alto, CA, USA. We used descriptive statistics for continuous distribution variables (averages and standard deviations) and statistic relevance was tested using Student’s *t*-test. To determine the statistical significance of the correlations existing between qualitative variables, we used the Pearson Chi-square value, calculating the Pearson-*r* coefficient and drawing up a graphic representation of the regression line. The associations between qualitative variables and continuous numeric variables were analysed using the variant analysis method (the ANOVA test). The predictive accuracy for the prognostic role of Gc-globulin serum level was assessed using receiver operating characteristic (ROC) curves, area under curve (AUC) values, confidence intervals and cut-off values for Gc-globulin using the easyROC program [21]. The cut-off value was selected depending on the Youden Index, which is considered the maximum potential effectiveness index for a biomarker’s ability to classify disease status. An AUC of 0.7–0.8 and greater than 0.8 were considered to indicate satisfactory and good accuracy of the proposed test, respectively [22]. In all analyses, the results were considered significant at *p* < 0.05. For *p* values < 0.01, we considered the test to have a good statistical significance, while *p* < 0.001 indicated that the statistical significance was extremely important (with an error margin of 0.1%).

## 3. Results

The ALF patients were aged between 1 month and 17 years and 10 months, with an average age of 4.31 ± 4.89 years (32 out of the 34 subjects were younger than 12 years old). The children included in the control group were aged between 6 months and 17 years and 10 months, with an average age of 6.11 ± 4.26 years (*p* = 0.124231). The ALF group comprised 18 boys (52.94%), as did the control group (60%, Pearson Chi-square 0.3226891, *p* = 0.5700).

The aetiology of ALF in our study group was metabolic disorder (9 patients, 26.47%), autoimmune disease (8 patients, 23.53%), toxic causes (7 patients, 20.59%), infection (6 patients, 17.65%) and unknown cause (4 patients, 11.76%). The evolution of the ALF patients included the death of 13 patients (38.24%), one patient who underwent a liver transplant (2.94%) and 20 patients who survived with supportive therapy (58.82%).

The characteristics and results of the lab tests for the children with ALF and the control group are presented in Table 1.

We analysed the different parameters in ALF patients depending on their evolution. The transplanted patient was included in the same analysis group as the deceased patients, since there was no survival of the native liver with supportive therapy. These data are presented in Table 2. 

Gc-globulin serum levels were significantly decreased in ALF patients (151.57 ± 171.87 mg/L, *p* < 0.000001) compared to the control group (498.63 ± 252.50 mg/L, which falls within the normal range of 350–500 mg/L) (Table 1 and Table 2) [12].

The surviving ALF patients had a mean serum level below the normal lower limit (216.12 ± 199.69 mg/L), while those with fatal evolution exhibited Gc-globulin values way below the normal limit (59.34 ± 33.73 mg/L, *p* = 0.006763). Extreme Gc-globulin levels were observed in both the reference and surviving ALF patients. With the exclusion of these cases from our analysis, the comparisons were still significant regarding the Gc-globulin serum levels.

INR levels, coagulation factors and Gc-globulin serum levels varied depending on the ALF aetiology. Gc-globulin serum levels were significantly lower in patients suffering from ALF due to metabolic disorder (42.54 ± 36.79 mg/L), infection (82.10 ± 14.11 mg/L) or unknown cause (87.89 ± 40.52 mg/L) compared to autoimmune (290.45 ± 200.40 mg/L) and toxic ALF (228.94 ± 234.02 mg/L) (*p* = 0.0102). Regardless of the cause of the ALF, the value of this parameter was lower overall in the ALF patients compared to the control group, where the average level was 498.63 ± 252.50 mg/L.

The correlation of Gc-globulin serum level with the other parameters already used in practice to assess the severity and prognosis of ALF was analysed, with Gc-globulin level showing a moderate–strong positive relationship with the levels of factor V (*r* = 0.570136; *p* = 0.001538) and factor VII (*r* = 0.582223; *p* = 0.000900), and a weaker negative relationship with the PELD score (*r* = −0.426294; *p* = 0.023694). There was also a weak positive correlation with albumin level (*r* = 0.357139; *p* = 0.062082) and a negative association with INR (*r* = −0.373167; *p* = 0.050477).

**Figure 1:**
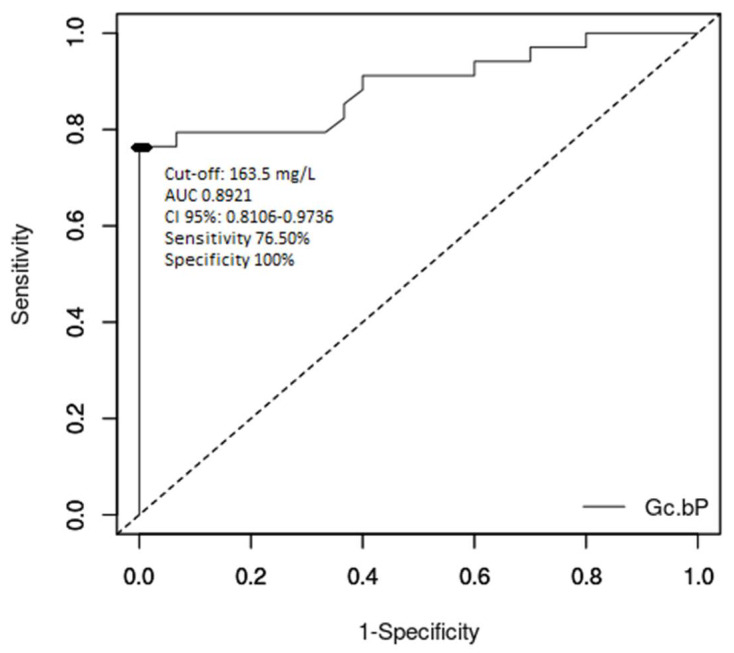
Receiver operating characteristics (ROC) curve for diagnosis of acute liver failure vs. healthy controls using serum levels of Gc-globulin.

To analyse the cut-off values of serum Gc-globulin in classifying ALF patients, including those with fatal evolution, we analysed area under curve (AUC) and receiver operating characteristic (ROC) curve values. Figure 1 presents the ROC curve for serum Gc-globulin levels in ALF patients compared to healthy subjects. The AUC value was 0.8921 (CI 95%: 0.8106–0.9736, *p* < 0.000001), while the optimum cut-off was 163.5 mg/L with a sensitivity of 76.50%, a specificity of 100%, a positive predictive value of 100% and a negative predictive value of 78.90%.

**Figure 2:**
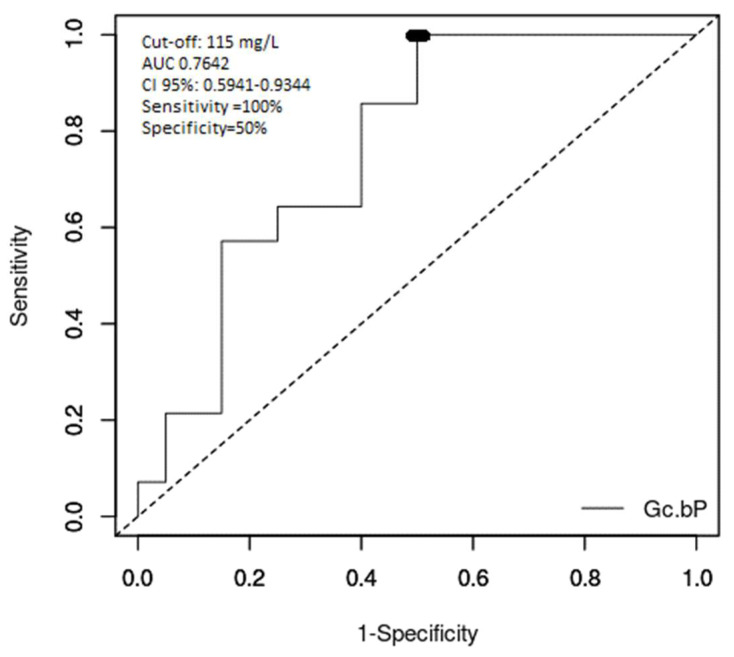
ROC curve for analysis of the outcome of acute liver failure in children using Gc-globulin serum levels.

Figure 2 shows the ROC curve analysis of the role of Gc-globulin in differentiating deceased patients. The AUC was 0.7642 (CI 95%: 0.5941–0.9344, *p* = 0.002329) and the optimum cut-off value for fatal evolution of ALF was 115 mg/L, with a sensitivity of 100%, a specificity of 50%, a positive predictive value of 58.30% and a negative predictive value of 100%.

The analysis of the King’s College criteria in ALF children included in our study revealed that these criteria were positive in seven patients with fatal evolution (7/14) and in one who survived (1/20). Thus, the sensitivity of King’s College criteria was 50% and the specificity was 95% with a positive predictive value of 87.50% and a negative predictive value of 73.10%. The accuracy of King’s College criteria in our patients was 76.47%.

## 4. Discussion

Acute liver failure (ALF) in children is associated with a high mortality rate (up to 50%) if a liver transplant is not urgently performed [7,8,9]. Various markers or scores are used to predict ALF outcomes. Extended prothrombin time (>50 s), low levels of plasma coagulation factors V and VII, cerebral oedema, severity of encephalopathy, sudden reduction in transaminases or liver size, high levels of α-fetoprotein (AFP), electrolyte disturbances, MELD and PELD scores and King’s College criteria were studied as predictive factors for the evolution of ALF in children [4,5,6,9,10,11,23,24,25,26,27]. Clichy–Villejuif Criteria, with factor V levels as the main parameter, are used to decide the need for emergency liver transplant in ALF [5,7,8,9]. However, none of these factors accurately predict possible negative evolution of ALF patients [6,7,28,29,30,31,32]; new markers should be studied to earlier predict the evolution of liver cell injuries.

Gc-globulin (also known as vitamin D-binding protein) is a serum α2-globulin with a molecular weight of 52–59 kDa, which was described first by Hirschfeld in 1959 [33]. Gc-globulin is encoded by the DBP gene, located on the long arm of chromosome 4 (4q12-q13); this gene encodes the entire protein family, comprising also albumin and α-fetoprotein [16,34]. Normal serum Gc-globulin levels are 350–500 mg/L [16]. Gc-globulin is synthesised by the liver and its role is to bind and transport vitamin D and its metabolites to target organs. It is also involved in the inflammatory response and, together with factor Va, triggers the process of disseminated intravascular coagulation (DIC) [16,17,18,35].

In our study carried out on 34 children with ALF of various aetiologies, we assessed the serum levels of Gc-globulin in these children compared to the control group and correlated these levels with ALF outcomes and other severity-associated disease parameters. Gc-globulin serum levels were much lower in ALF patients compared to the control group, most likely a secondary effect of the aggression directed toward the liver, thereby affecting the hepatic synthesis processes. The average Gc-globulin serum level was also lower in patients with poor outcomes compared to those who survived (Table 2).

Only a few studies are available in the literature describing total Gc-globulin or actin-free Gc-globulin level reductions in cases of acute liver injury, and these works focused on adults [34,35,36,37,38,39,40]. The first reports of Gc-globulin level changes in ALF patients were published by Lee, Galbraith, and Goldschmidt-Clermont [17,18,19]. Schiødt also described a drop in Gc-globulin levels in patients suffering from acute or chronic liver disease and normalization once the patients underwent liver transplantation. According to Schiødt, adult patients with fatal evolution of ALF showed significant drops in Gc-globulin levels on admission compared to patients who survived. The predictive cut-off value of 100 mg/L was equal to King’s College criteria in the prediction of the outcome [34,38,39,40]. The use of Gc-globulin as a prognostic marker of ALF outcomes was also described in a study carried out in the United States on 106 patients with non-paracetamol ALF. In this study, serum Gc-globulin levels under 80 mg/L and King’s College criteria both demonstrated high positive predictive values but low negative predictive values. [16]

In our study, Gc-globulin levels in ALF varied with the aetiology. Gc-globulin levels were lower in ALF caused by metabolic, infectious, or unknown factors (possibly infection) compared to those caused by autoimmune and toxic factors, possibly due to greater severity of hepatic necrosis. In autoimmune ALF, even though the severity of the disease and the outcome could be poor, chronic evolution and the inflammatory process could explain the higher levels of Gc-globulin. This was also reported by studies performed in adults, where Gc-globulin levels were significantly lower in ALF patients and less so in those with chronic disease or chronic liver failure, such as autoimmune liver disease [34]. Also, Gc-globulin levels were significantly higher in patients with paracetamol-induced ALF compared to other causes of ALF [16,39]. The explanation of this difference was linked to the prognosis shown in cases of paracetamol-induced ALF, where spontaneous chances of survival are higher than 50%, compared to non-paracetamol ALF, where survival probability is lower than 25% [16,17,37,39].

We also analysed the correlation between Gc-globulin and other parameters used for the prognosis of ALF and the PELD score. Low serum levels of Gc-globulin were highly significantly associated with low serum levels of factor V and factor VII and a high PELD score, which all indicate severe liver injury and a negative prognosis. No statistically significant correlation was revealed for albumin, which could be explained by the fact that Gc-globulin levels usually drop faster than albumin. This lack of correlation between the two related proteins, which are encoded by the same gene, was also revealed by Schiødt in patients who underwent liver transplantation.

Our study showed an overall good performance of Gc-globulin in differentiating ALF in children (AUC 0.8921), with an optimum cut-off value of 163.5 mg/L. The performance of serum levels of Gc-globulin to differentiate children at risk for poor ALF outcomes was moderate (AUC 0.7642). The cut-off value for poor outcome without liver transplantation was 115 mg/L. Compared to the King’s College criteria, this cut-off value of serum Gc-globulin provides better sensitivity, but lower specificity and positive predictive value. When comparing this result to the literature, this cut-off was higher than in ALF in adults (80 mg/L) [40]. Antoniades reported that actin-free Gc-globulin levels were reduced by 90% in ALF and 70% in cirrhotic adult patients [40], which was related to the severity of liver dysfunction and the inflammatory response (correlations with acidosis, coagulopathy and encephalopathy). In this study, a cut-off level of 46.5 mg/L was 76% sensitive and 61% specific as a prognostic factor of poor outcome. The AUC was 0.7, which was lower than for arterial blood lactate, the severity of the encephalopathy, INR and the Sequential Organ Failure Assessment (SOFA) score and higher than norepinephrine, the Acute Physiology and Chronic Health Evaluation (APACHE) II score and bilirubin [41]. Thus, knowing that the currently used criteria have some limitations, Gc-globulin can be considered as a prognostic marker for severe evolutions and indication for liver transplantation.

The main limitation of our study was the small number of children with ALF included in the sample population, as this condition is rare in the paediatric population. Moreover, a larger number of ALF patients from different causes would increase the accuracy of the trend anaylsis of Gc-globulin in different etiologies. Still, this work constitutes one of only a few studies assessing Gc-globulin or other markers for the evaluation of the severity of ALF in children.

## 5. Conclusions

In summary, our results confirmed in children the already existing data for adults regarding decreased serum levels of Gc-globulin in ALF and the correlations with the severity of liver injury. Gc-globulin serum levels could help to identify patients at high risk for poor outcomes. Additional studies are needed to further detail the prognosis potential of Gc-globulin serum levels in ALF in children.

## Figures and Tables

**Table 1:** Comparison between children with acute liver failure (ALF) and control group (non-ALF).

Variable	ALF (*n* = 34)	Controls (*n* = 30)	*p* Value
Age (years)	4.31 ± 4.89	6.11 ± 4.26	0.124231
Sex: Males	18 (52.94%)	18 (60%)	0.5700
Aetiology of ALF:			-
Metabolic disorders	9 (26.47%)	-	
Autoimmune hepatitis	8 (23.53%)	-	
Toxic	7 (20.59%)	-	
Infections	6 (17.65%)	-	
Unknown	4 (11.76%)	-	
AST (UI/dL)	1466.53 ± 2203.44	32.77 ± 8.02	0.000717
ALT (UI/dL)	1265.24 ± 1517.38	20.03 ± 8.17	0.000032
Factor V (%)	37.25 ± 26.75	86.62 ± 16.11	<0.000001
Factor VII (%)	25.74 ± 24.52	87.56 ± 21.51	<0.000001
INR	4.63 ± 3.17	1.29 ± 0.21	<0.000001
% Prothrombin	29.23 ± 20.81	83.84 ± 19.05	<0.000001
Gc-globulin (mg/L)	151.57 ± 171.87	498.63 ± 252.50	<0.000001
PELD score	22.57 ± 13.94	-	-
Evolution:			-
Alive	20 (58.82%)	30	
Liver transplantation	1 (2.94%)	-	
Deceased	13 (38.24%)	-	

Data are given as mean ± standard deviation (SD) or number of cases (percentage). ALF: acute liver failure; AST: aspartate transaminase; ALT: alanine transaminase; INR: International Normalised Ratio; PELD: Paediatric End-stage Liver Disease.

**Table 2:** Comparison of different parameters depending on the outcome of acute liver failure.

Parameters	Deceased or Liver Transplanted (*n* = 14)	Alive (*n* = 20)	*p* Value
AST (UI/dL)	1406.71 ± 1749.22	1508.40 ± 2516.80	0.897028
ALT (UI/dL)	1198.43 ± 1680.04	1312.00 ± 1436.04	0.833716
Total Bilirubin (mg/dL)	8.83 ± 7.71	7.07 ± 7.22	0.503015
Direct Bilirubin (mg/dL)	5.50 ± 4.26	5.36 ± 5.88	0.937538
Factor V (%)	18.93 ± 19.96	43.90 ± 26.98	0.008193
Factor VII (%)	10.72 ± 10.22	38.89 ± 26.02	0.000724
INR	7.06 ± 3.20	2.93 ± 1.75	0.000031
% Prothrombin	14.86 ± 14.06	39.29 ± 18.92	0.000267
Gc-globulin (mg/L)	59.34 ± 33.73	216.12 ± 199.69	0.006763
PELD score	30.06 ± 15.55	16.76 ± 9.35	0.005336

Data are given as mean ± standard deviation (SD). AST: aspartate transaminase; ALT: alanine transaminase; INR: International Normalised Ratio; PELD: Paediatric End-stage Liver Disease.
